# Numerical Demonstration of In-Tube Liquid-Column Migration Driven by Photoisomerization

**DOI:** 10.3390/mi9100533

**Published:** 2018-10-20

**Authors:** Kei Nitta, Takahiro Tsukahara

**Affiliations:** 1 Department of Mechanical Engineering, Tokyo University of Science, 2641 Yamazaki, Noda, Chiba 278-8510, Japan; 7517651@ed.tus.ac.jp; 2 Water Frontier Science & Technology Research Center (W-FST), Research Institute for Science & Technology, Tokyo University of Science, 1-3 Kagurazaka, Shinjuku-ku, Tokyo 162-8601, Japan

**Keywords:** computational fluid dynamics, droplet manipulation, lab-on-a-chip, microfluidics, non-invasive control, photochemical reaction, photoresponsible surfactant, surface tension, two-phase flow, wettability

## Abstract

Droplet manipulation by light-induced isomerization was numerically demonstrated and investigated regarding the driving mechanism. Such a non-invasive manipulation of a droplet in a microchannel can be realized, for example, by the use of watery solution of photoresponsive surfactant that exhibits the isomerization. Due to variable fluid properties between the *cis* and *trans* isomers, one-side light irradiation on a liquid column in a tube would lead to some kind of imbalance between the two ends of the liquid column and then drive droplet migration. The present numerical simulations of air–liquid two-phase flow and its scalar transport of the isomer, considering the variable static contact angle, agreed quantitatively with the experimental results in terms of the migration speed. This fact supports the contention that the droplet migration is more likely to be driven by an imbalance in the wettability, or the contact angle. The migration speed was found to be less dependent on the liquid-column length, but proportional to the tube diameter.

## 1. Introduction

Droplet manipulation techniques for microfluidic devices and lab-on-a-chip have attracted much attention in various fields such as medicine, chemistry, and biology [1,2,3,4,5]. This is because such an analytic operation on a micro scale increases the surface-to-volume ratio, thus obtaining many advantages, for instance, minituarization of samples, high-speed reaction, and downsizing of devices. Hence, several researches have devised a method of fluid driving in a micro-scale channel by changing the surface tension and wettability, e.g., a plasma-etched polymer nanostructure that enhances the droplet mobility [6], dielectrophoresis that employs an electric dipole moment by immersing an electrocode in a channel [7], and EWOD (Electrowetting on Dielectric) that generates the wettability gradient due to static electricity [8]. However, the challenges related to those methods include not only the fabrication of the channel but also the contamination by contact and the difficultly of flexible and selective manipulation. An alternative method using light as an external stimulus has several advantages such as not requiring the fabrication of a channel, simple adjustment of the stimulus, flexible and selective manipulation, and less contamination due to the non-contact [9]. In addition, it can be utilized on a living body due to its non-invasiveness.

There exist various ways of driving liquid by light irradiation: optical tweezers [10,11], photothermal Marangoni flow [12,13,14,15], and photoisomerization [16,17,18,19,20,21,22,23]. Optical tweezers is a method that uses the radiation pressure of light, but its corresponding force is very small, in the order of pico-newtons. The use of photothermal Marangoni flows should be accompanied by heat, together with the light irradiation. This method is flexible and selectively operable, and the resulting force is sufficient for manipulation; however, it cannot be applied to a substance that is denatured by heat. On the other hand, the fluid manipulation by utilizing *cis-trans* photoisomerization can avoid the use of heat, although it is necessary to change the fluid properties in response to light.

**Figure 1:**
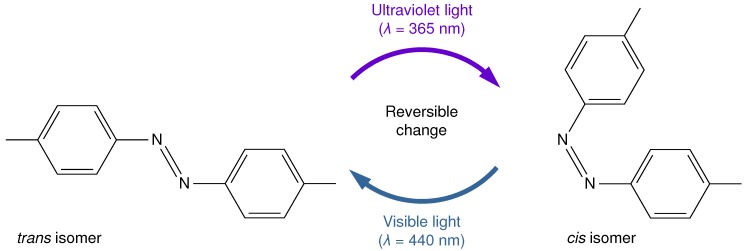
Schematic diagram of the photochemical isomerization, reproduced on the basis of Ref. [23]. The *cis* isomer irradiated with visible light should change into the *trans* isomer, while the *trans* isomer irradiated with ultraviolet (UV) light would change into the *cis* isomer. These two isomers may exhibit different fluid properties. The rate at which the number of isomers increases or decreases by photo-induced isomerization is expressed as a reaction rate constant *k*.

As shown in Figure 1, the *cis-trans* photoisomerization is a property in which the *cis* and *trans* isomers are reversibly changed by light of a specific wavelength such as ultraviolet (UV) light [24]. The isomers are represented by the same molecular formula but different molecular structures. As the molecular structure changes, fluid properties such as the contact angle and surface tension are varied. Here, the reaction rate constant *k* represents a rate at which the reactants increase or decrease in a chemical reaction. In the photoisomerization, *k* represents the degree of photo-induced change between the *cis* isomer and the *trans* isomer. Azobenzene is a representative example [25,26,27,28]. In previous studies, droplets on a substrate were manipulated by performing photoisomerization on the substrate and changing wettability [16,17,18,19,20], or on photoresponsive surfactant [20,21,22,23]. Recently, Muto et al. [23] demonstrated the manipulation of a liquid column in a millimeter-scale glass tube. Its droplet manipulation was done by UV-light irradiation on a one-side surface of the liquid column generating differences in the contact angle and surface tension between both sides of the finite liquid column. The mobility of the migrated liquid column was reported to depend on the liquid column length. However, such an experimental demonstration might often suffer from a pinning effect that cannot be avoided, and its flow dynamics and developments of each isomer distribution are not fully understood, thus increasing the difficulty of experimental measurement. To the authors’ knowledge, no numerical simulation of such a liquid driving has been performed.

In order to reveal the light-induced droplet-migration phenomenon due to *cis*-*trans* isomerization in a watery solution of photoresponsive surfactant, we carried out a numerical study of in-tube liquid columns, considering the variable contact angle and surface tension. We used a framework of OpenFOAM${}^{®}$ (version 2.3.1) [29], which is an open source software and has been verified by benchmark tests for multiphase flows [30,31,32,33,34]. The Volume-of-Fluid (VoF) method [35] is used as an interface-capturing method, and the Continuum-Surface-Force (CSF) model [36] is applied to calculate the surface tension on liquid–air interfaces. In addition, we have implemented the Continuous-Species-Transfer (CST) method [37] to express the *cis*/*trans*-isomer transports more accurately. We discuss a validation with the experiment [23], and numerically investigate the isomer distribution and the effects of the liquid column length and radius on the liquid-column migration.

## 2. Problem Setting: A Photoisomerizable Liquid Column in a Tube

**Figure 2:**
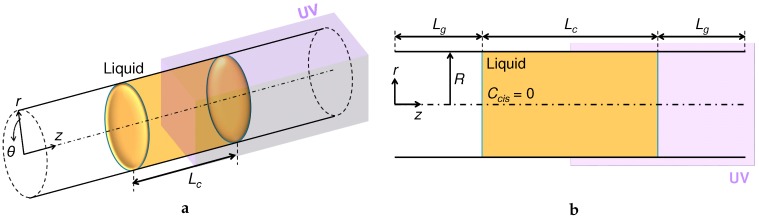
Coordinate system and configuration of an in-tube photoisomerizable liquid column to be irradiated partially with UV light. (**a**) Bird’s eye view; (**b**) cross-sectional view. The liquid column is initially located at the center of the tube domain and in the form of a simple column shape, being surrounded by air. The length of the liquid column is denoted as $L_{c}$, while that of the gas phase is $L_{g}$.

We focus on an experimental demonstration performed by Muto et al. [23] and employ a similar problem setting for our numerical analysis. Figure 2 shows the present analysis object, which is a liquid column given in an infinite straight tube with a constant radius *R*. The liquid column was initially placed at the center of the computational domain. A UV light is assumed to be irradiated on the right half of the domain: see Figure 2. The irradiation is started from the state of all *trans* isomers in the liquid of interest; that is, the initial $C_{cis}$ was 0 in the entire domain. Since the tube is on the millimeter-scale, as tested by Muto et al. [23], the UV light is assumed not to decay throughout the liquid [38]. In the simulation, we used a wedge mesh which consisted of a single grid cell in the circumferential direction by assuming an axisymmetric flow with respect to the *z* axis. This allows us to reduce the computational cost by keeping fine meshes in the other directions. While Muto et al. [23] used a sufficiently-long open tube for their measurement, it is practically difficult to simulate such a system rigorously. Two different boundary conditions in the *z* direction were tested in this study: the periodic boundary condition (PBC) and the inlet/outlet boundary condition. On the tube surface, the no-slip boundary condition was applied. The initial length of the gas phase on both sides of the liquid column was set at $L_{g}=15$ mm, regardless of the liquid column length $L_{c}$.

Table 1 shows the fluid properties of our present targets. Since we simulated both the air and liquid phases simultaneously, the air properties at room temperature were given. Essentially important properties in the present problem are the contact angle ϑ and the surface tension σ, as explained in Section 1. Those values vary depending on the isomer, and the liquid of our interest reveals rather hydrophilic features with the *cis*-isomer: the contact angle of purely *cis*-isomer liquid, $\vartheta_{cis}$, is slightly lower than that for the *trans*-isomer, $\vartheta_{trans}$. We referred to the values of $\vartheta_{cis}$ and $\vartheta_{trans}$ measured experimentally by the extension/contraction method, and $\sigma_{cis}$ and $\sigma_{trans}$ measured by the pendant drop method [39]. The reaction rate constant *k* for the photoisomerization was identified by ${}^{1}$H-NMR measurement. The concentration diffusivity *D* for the isomer diffusion in each fluid was chosen as a typical value: cf., Ref. [28].

## 3. Numerical Procedure

### 3.1. Governing Equations for Fluid Motions

Although the actual system of our interest consists of incompressible liquid and compressible air contained in a tube, we considered the air phase as incompressible for simulating the fluid behavior. This assumption allows us to use the governing equations of incompressible and immiscible gas–liquid two-phase flows: the equation of continuity

(1) ∇·u=0

and the Navier–Stokes equation

(2) $$\rho \left\{\partial_{t}u+\left(u\cdot \nabla \right)u\right\}=-\nabla p+\mu \nabla^{2}u+f_{\sigma },$$

which includes a surface tension force $f_{\sigma }$ that works on the liquid–air interfaces based on the Continuum-Surface-Force (CSF) model [36]. In the present study, all simulations were under the zero-gravity condition. We used the Volume-of-Fluid (VoF) method [35], which is a well-known way to capture an interface between two different fluids. In this method, the advection equation of the VoF function α can be written as

(3) $$\partial_{t}\alpha +\left(u\cdot \nabla \right)\alpha =0.$$

The VoF function α describes the liquid fraction in each computational grid cell to determine the two-fluid allocation. In the present air–liquid two-phase flow, we defined it as

(4) $$\begin{array}{c} \alpha =\left\{\begin{array}{cc} 1 & :\mathrm{Liquid}\;\mathrm{phase}\\ 0 & :\mathrm{Air}\;\mathrm{phase}\\ \left(0,1\right) & :\mathrm{Interface} \end{array}\right. \end{array}$$

Instead of using simply Equation (3), one may compute the following equation with an artificial term with the aim of sharpening the liquid–air interface.

(5) $$\partial_{t}\alpha +\left(u\cdot \nabla \right)\alpha +\nabla \cdot \left(\alpha \left(1-\alpha \right)u_{r}\right)=0.$$

The third term on the left hand side of Equation (5) represents the artificial interface compression term, in which the compression velocity $u_{r}$ is calculated, as follows:

(6) $$u_{r}=n_{f}\min\left(C_{\alpha }\left|\frac{\phi }{S_{f}}\right|,{\left|\frac{\phi }{S_{f}}\right|}_{\max}\right).$$

Here, $n_{f}$ is the normal vector of the interface in a grid-cell control volume, ϕ is the mass flux, $S_{f}$ is the area of the interface in the control volume, and $C_{\alpha }$ is an adjustable coefficient. While $C_{\alpha }$ can be changed arbitrary, we decided to set $C_{\alpha }=0$ in this study to make unavoidable spurious currents less pronounced, as discussed in Appendix A.1.

The local density ρ and viscosity μ at each grid cell depend on α:

(7) $$\rho =\rho_{l}\alpha +\rho_{g}\left(1-\alpha \right),$$

(8) $$\mu =\mu_{l}\alpha +\mu_{g}\left(1-\alpha \right).$$

The surface tension term in Equation (2) is expressed as

(9) $$f_{\sigma }=\sigma \kappa \nabla \alpha$$

in the CSF model [36]. The unit normal vector n and the curvature κ of a local interface can be expressed by the VoF function α, respectively, as

(10) $$n=\frac{\nabla \alpha }{\left|\nabla \alpha \right|},\;\kappa =-\nabla \cdot n.$$

Note here that Equation (9) includes only the normal force on the liquid–air interface, neglecting the tangential force. The normal force induces the Laplace pressure, while the Marangoni convection may be triggered by tangential force on the interface. Compared to the normal force, the expected tangential force would be much smaller in this study, because the difference between $\sigma_{cis}$ and $\sigma_{trans}$ is almost negligible relative to its absolute value, as given in Table 1.

### 3.2. Representation of Cis-/Trans-Isomer Liquid

Since the driving force exerted on a liquid column by the *cis-trans* photoisomerization is induced by the imbalance of the surface tension and contact angle (or Laplace pressure), it is necessary to express numerically the two different states of either the *cis* or *trans* isomer and to model the exchange between them due to the photoisomerization. Then, let us introduce $C_{cis}$, which is defined in each computational grid cell as the volume–fraction ratio of the *cis* isomer:

(11) $$\begin{array}{c} C_{cis}=\left\{\begin{array}{cc} 1 & :\mathrm{All}\;\mathrm{cis}\;\mathrm{isomer}\\ 0 & :\mathrm{All}\;\mathrm{trans}\;\mathrm{isomer}\\ \left(0,1\right) & :\mathrm{Mix}\;\mathrm{of}\;\mathrm{cis}\;\&\;\mathrm{trans}\;\mathrm{isomers} \end{array}\right. \end{array}$$

Both the contact angle and surface tension were determined as a function of $C_{cis}$:

(12) $$\vartheta =\vartheta_{cis}C_{cis}+\vartheta_{trans}\left(1-C_{cis}\right),$$

(13) $$\sigma =\sigma_{cis}C_{cis}+\sigma_{trans}\left(1-C_{cis}\right).$$

A validation test was performed on a simple droplet on the flat wall with a variable contact angle that depends on $C_{cis}$, and we confirmed a reasonable $C_{cis}$-dependence of the contact angle as well as a response of the droplet wetting to UV-light irradiation, as presented in Appendix A.2.

### 3.3. Transport of the Cis/Trans Isomer with the CST Method

The transport equation of the isomer ratio $C_{cis}$ can be written as follows:

(14) $$\partial_{t}C_{cis}+\left(u\cdot \nabla \right)C_{cis}=D\nabla^{2}C_{cis}+R,$$

where *R* is the source term due to the photochemical reaction and *D* is the arithmetic mean diffusivity:

(15) $$D=D_{l}\alpha +D_{g}\left(1-\alpha \right)$$

In the actual phenomenon, no transport of the isomer across the liquid–air interface should occur. However, unavoidable spurious currents, i.e., numerically-artificial flows, in simulation may lead to scalar transport across the interface. To suppress such a leakage of scalar (or $C_{cis}$ in this study) across the liquid–air interface, we implemented the Continuous-Species-Transfer (CST) method [37]. Then, Equation (14) is reformulated into

(16) $$\partial_{t}C_{cis}+\left(u\cdot \nabla \right)C_{cis}=\nabla \cdot \left(D\nabla C_{cis}+\Phi \right)+R,$$

where Φ is the discontinuity term and it works at the interface, as follows:

(17) $$\begin{array}{cc} \Phi = & -\left(D_{l}-D_{g}\right)\alpha \left(\frac{1}{\alpha +\left(1-\alpha \right)/H}\right)\\ & \;+\frac{C_{cis}}{\alpha +\left(1-\alpha \right)/H}\left\{\frac{1}{H}\frac{D_{l}-D_{g}}{\alpha +\left(1-\alpha \right)/H}-\left(D_{l}-\frac{D_{g}}{H}\right)\right\}\nabla \alpha \end{array}$$

The degree of suppression can be calibrated by the Henry coefficient *H*. We set H=100: see also Appendix A.1.

It should be noted that, in the present simulation, only the change from the *trans* to *cis* photoisomerization by UV-light irradiation was expressed by

(18) $$R=\alpha \left\{k\left(1-C_{cis}\right)\right\}\delta_{\mathrm{UV}},$$

but the opposite change from the *cis* to *trans* photoisomerization (by visible light) was not considered. Because the latter photoisomerization would not provide any contribution to the liquid-droplet migration under the present condition. Here, *k* in Equation (18) is the reaction rate constant of change from *trans* to *cis*, and $\delta_{\mathrm{UV}}$ is

(19) $$\begin{array}{c} \delta_{\mathrm{UV}}=\left\{\begin{array}{cc} 1 & :\mathrm{UV}-\mathrm{light}\;\mathrm{irradiation}\;\mathrm{part}\\ 0 & :\mathrm{Others} \end{array}\right. \end{array}$$

Equation (18) expresses that the UV-photoisomerization occurs only in a liquid portion that is irradiated with UV light, by multiplying α and $\delta_{\mathrm{UV}}$. Basically, $C_{cis}=0$ should be kept in the air and/or non-irradiated part.

## 4. Results

### 4.1. Grid Resolution: Comparative Validation with Experimental Results

**Figure 3:**
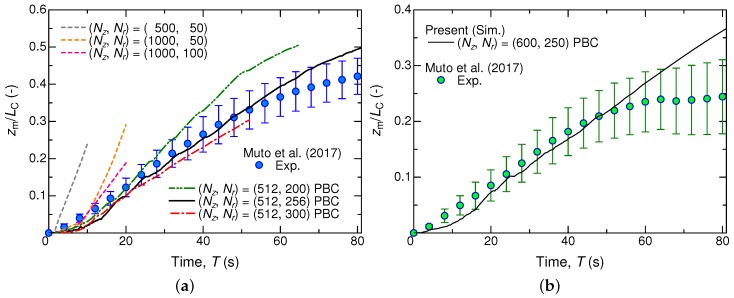
Temporal variations of the migration distance $z_{m}$ of the liquid column normalized by the liquid column length $L_{c}$, calculated by Equation (20), and comparison with the experimental results [23]. In the legend, ‘PBC’ represents a case with the periodic boundary condition in the *z* direction. (**a**) $L_{c}=20$ mm, (**b**) $L_{c}=30$ mm. The tube radius is R=1.25 mm.

Figure 3 shows our obtained migration distance $z_{m}$, normalized by the liquid column length $L_{c}$, from the initial position as a function of the irradiation time *T*. The instance of T=0 corresponds to the beginning of the simulation as well as that of the UV-light irradiation. The migration distance $z_{m}$ from the initial position $z_{0}$ was calculated from the axial shift of the center of gravity of the liquid column.

(20) $$z_{m}=\frac{\int_{V}\alpha \cdot zdV}{\int_{V}\alpha dV}-z_{0}$$

According to the experimental results shown also in Figure 3, the liquid column would be transported at a constant speed toward the UV light side (i.e., to the positive *z* direction). As $z_{m}/L_{c}$ approaches 0.5, the migration speed appears to decelerate and finally the liquid column stops moving. Note here that $z_{m}/L_{c}=0.5$ corresponds to the situation that the entire liquid column has just entered the UV area. To demonstrate such phenomena in our simulation, we first examined the required grid resolution, or the number of grids in the axial- ($N_{z}$) and radial- ($N_{r}$) directions. Our parametric study for $N_{z}=500$–2000 and $N_{r}=25$–300 revealed that a combination of $\left(N_{z},N_{r}\right)=\left(512,256\right)$ was required for the case of $L_{c}=20$ mm, as shown in Figure 3a. The linear motion in the initial stage could be reproduced even with lower resolutions, but the migration speed was overestimated remarkably. Furthermore, regarding the *z*-direction boundary condition, the periodic boundary condition (PBC) was found to provide a better result, compared to the inlet/outlet condition.

As for a longer liquid column of $L_{c}=30$ mm, we achieved good quantitative agreement with the experimental results, as shown in Figure 3b. Similar to the shorter case mentioned above, the liquid column exhibited an acceleration period until T<20 s, and it attained a constant migration speed at T≈20 s. Whereas the linear motion obviously terminated before reaching $z_{m}/L_{c}=0.5$ in the experiment, the present simulation shows a continuous linear motion until $z_{m}/L_{c}$ reaches 0.5. Such a deviation in this late stage of the droplet migration must be attributed to the pinning effect that cannot be simulated with the present code. At least, in the initial stage including the linear motion, the photoisomerization-induced droplet migration has been reasonably demonstrated by our simulation.

**Figure 4:**
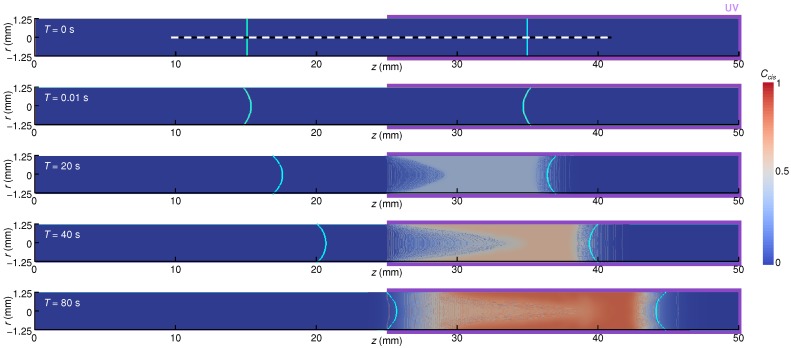
Cross-sectional views of the liquid column with a length of $L_{c}$ = 20 mm and the developing distribution of the ratio of the *cis* isomer, $C_{cis}$, during the droplet migration driven by photoisomerization by a partial UV-light irradiation, at time instances of 0, 0.01, 20, 40, and 80 s (from top to bottom). The tube radius is R=1.25 mm. Contour shows the ratio of the *cis* isomer: $C_{cis}=1$, all *cis* isomers; $C_{cis}=0$, all *trans* isomers. Cyan solid lines indicate the liquid–air interface positions; purple-colored backgrounds (on the right half of the domain) represent the UV-light irradiated parts.

**Figure 5:**
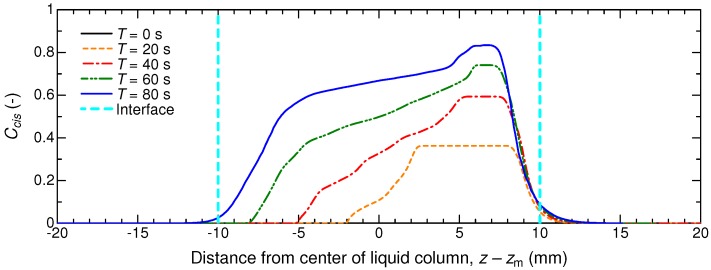
Temporal variation of $C_{cis}$ along the white dashed line in Figure 4 for $L_{c}=20$ mm. The horizontal axis represents the relative position with respect to the center of the liquid column. The interface on the UV-light irradiation side is located at $z-z_{m}\approx 10$ mm, and the opposite one is at $z-z_{m}\approx -10$ mm.

Figure 4 shows the developing distribution of $C_{cis}$ in the *z*-*r* section of the domain including both the air and liquid phases. The top panel in the figure represents the initial condition. At T=0.01 s, the liquid–air interface is already concave due to the wettability with $\vartheta <90^{\circ }$ and surface tension. This implies that the timescale of surface deformation is much faster than the reaction rate of the photoisomerization of interest. From T=20 s, one can recognize that the isomers in the UV-light irradiation side changed gradually from *trans* to *cis* with time, but it can also be recognized that an axial shift of the liquid column already occurred. Figure 5 shows the temporal variation of the distribution of $C_{cis}$ on the *z*-axis inside the liquid column, which is marked with a white dashed line in Figure 4. The horizontal axis represents the *z* subtracted by the moving distance $z_{m}$ of the liquid column: $z-z_{m}=0$ corresponds to the center of the liquid column and the air–liquid interfaces locate at about $z-z_{m}=\pm 10$ mm because $L_{c}=20$ mm. As seen in the figure, $C_{cis}$ increases firstly on the UV-light irradiation side because of the photoisomerization. As time progresses, the plateau of the $C_{cis}$ profile becomes high and narrow ($z-z_{m}=2$–8 →6–7 as T=20→80), while the values gradually start to increase also in the column that is half on the non-irradiated side. This is the result of competition between the photoisomerization reaction and the incoming *trans*-isomer liquid from the non-irradiated side. At T=80 s, the non-zero $C_{cis}$ region reaches the interface on the non-irradiated side. This is to be expected due to the fact that the liquid column starts to decelerate its migration at about T=80 s. From this, we may draw the conclusion that the liquid column would stop when the *cis* isomer concentrations at both sides of the liquid column are comparable.

### 4.2. Influence of the Liquid Column Length and Tube Radius

**Figure 6:**
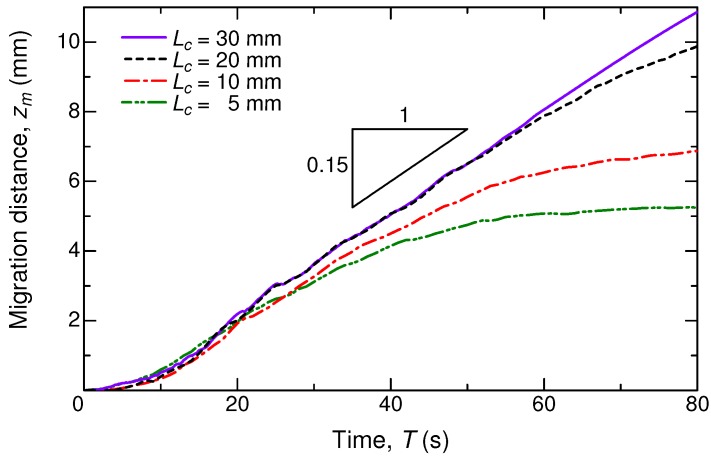
Time series of the migration distance of the liquid column for different liquid column lengths, without normalization. With R=1.25 mm, $N_{z}=350$–600, and $N_{r}=256$.

Furthermore, the dependence of the migration motion on the column length $L_{c}$ was investigated. Four column lengths of 5, 10, 20, and 30 mm were examined under the same system with a fixed tube radius R=1.25 mm, and the initial exposed column length was set to $L_{c}/2$ for all cases. Figure 6 compares the four cases, where the profiles are plotted in a dimensional form of millimeter *versus* second. From the gradient of each profile, the migration speed can be determined. The present numerical results obviously reveal the independence of the migration speed on the column length, at least in the initial stage of the migration. This aspect contradicts the experimental observation [23], but this mismatch might be due to a pinning effect on an actual tube in the experiment. With the increasing $L_{c}$, the migration distance is elongated further. The constant linear motion is found to be at a speed of ≈0.15 mm/s, irrespective of $L_{c}$. This speed could be varied by changing the fluid properties as well as the tube geometry. Further investigation on the fluid-property dependence of the migration speed is potentially interesting for a practical application of the photoisomerization in a droplet manipulation.

**Figure 7:**
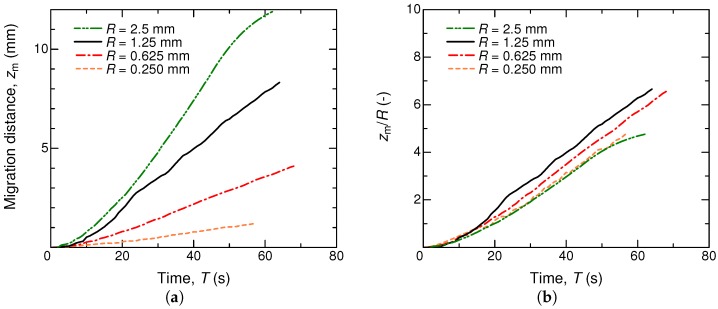
Time series of the migration distance of the liquid column for different tube radii in units of millimeter (**a**). In (**b**), the migration distance is normalized by the tube radius. With $L_{c}=20$ mm, $N_{z}=512$, and $N_{r}=256$.

The dependence on the tube radius was not investigated experimentally [23], so we numerically examined it under the same fluid conditions but with various *R*. Figure 7 shows results for R=0.25–2.5 mm. The temporal evolution of the migration distance did not change qualitatively with a different *R*, but the migration speed was found to vary significantly. Normalized by each tube radius, the profiles are scaled well, as given in Figure 7b. According to the present numerical result, we conjecture that the migration speed is roughly proportional to the tube radius. This aspect is consistent with Muto et al. [23] with respect to their discussion on the net driving force *F* that was exerted on the liquid column. They suggested a form of

(21) $$F=2\pi R\left(\sigma_{cis}\cos\vartheta_{a\text{-}\mathrm{cis}}-\sigma_{trans}\cos\vartheta_{r\text{-}\mathrm{trans}}\right),$$

where $\vartheta_{a\text{-}\mathrm{cis}}$ and $\vartheta_{r\text{-}\mathrm{trans}}$ denote the advancing contact angle of the *cis* isomer and the receding one of the *trans* isomer, respectively. Actually, Equation (21) does not relate to the column length $L_{c}$, but includes the tube radius *R*, supporting also the $L_{c}$-independency observed in Figure 6.

Last, but not least, we would argue that the main driving force of the liquid-column migration by photoisomerization is attributed to the difference between the static contact angles $\vartheta_{\mathrm{cis}}$ and $\vartheta_{\mathrm{trans}}$. In the context of Equation (21), the given condition of $\vartheta_{\mathrm{cis}}<\vartheta_{\mathrm{trans}}$ reasonably results in F>0. In terms of the surface tension, $\sigma_{cis}$ that is slightly larger than $\sigma_{trans}$ might also contribute to the onset of a positive *F*, but a comparison such as $\cos\vartheta_{\mathrm{cis}}/\cos\vartheta_{\mathrm{trans}}>\sigma_{cis}/\sigma_{trans}$ (cf. Table 1) implies that the contact-angle variation would account for the imbalance between the two ends of the liquid column. On the other hand, Muto et al. [23] declared that the migration was induced by a difference in the surface tension rather than the contact angle, because they observed $\vartheta_{a\text{-}\mathrm{cis}}\ll \vartheta_{r\text{-}\mathrm{trans}}$; that is, $\cos\vartheta_{a\text{-}\mathrm{cis}}<\cos\vartheta_{r\text{-}\mathrm{trans}}$ and this fact should demand $\sigma_{cis}\gg \sigma_{trans}$ to derive the positive *F* from Equation (21). However, they still suffered from difficulties in measuring the dynamics contact angle and surface tension and did not perform quantitative evaluation. Although our conclusion opposes their hypothesis, the quantitative agreement in the migration speed with the experimental result supports the present conclusion that the wettability, or the contact angle, is key to the driving force for the present droplet manipulation.

## 5. Conclusions

We performed numerical simulations to investigate the liquid-column migration driven by the *cis*-*trans* photoisomerization phenomenon that was experimentally demonstrated by Muto et al. [23]. The liquid-column migration should be induced by an imbalance in the wettability, or the contact angle and surface tension, between the two ends of the liquid column. To track the liquid–air interface, we employed the VoF method in conjunction with the CST model and the CSF model. In order to express the developing distribution of the *cis*/*trans* isomer, we also defined a volume–fraction ratio of the *cis* isomer, on which both the contact angle and surface tension were dependent. Neglecting the gravity, evaporation, thermocapillary, and dynamic contact angle, our simulation successfully demonstrated the photoisomerization-induced droplet migration and achieved good agreement with the experimental results. Our conclusion regarding the mechanism of the present droplet manipulation is that the driving force is caused mainly by the imbalance in the wettability, or the contact angle, between the two ends of liquid column rather than the surface tension. Through a numerical investigation of the *cis* isomer distribution, which is difficult to measure experimentally, we confirmed that the liquid-column migration terminated when the *cis* isomer distribution reached the non-irradiated region. We also found that the migration speed was less dependent on the liquid-column length and was proportional to the tube diameter.

## Figures and Tables

**Table 1:** Fluid properties: ρ, density; μ, viscosity; *D*, mass concentration diffusivity; ϑ, static contact angle on the solid wall; σ, surface tension; and *k*, reaction rate constant.

ρ (kg/m${}^{3}$)			μ (mPa·s)			*D* (m${}^{2}$/s)			ϑ (${}^{\circ }$)			σ (mN/m)			*k* (s${}^{-1}$)
Liquid	Air		Liquid	Air		Liquid	Air		*Cis*	*Trans*		*Cis*	*Trans*		
992.3	1.247		0.890	0.018		$10^{-9}$	$10^{-5}$		31.4	36.6		41.0	40.0		0.0225

**Figure A1:**
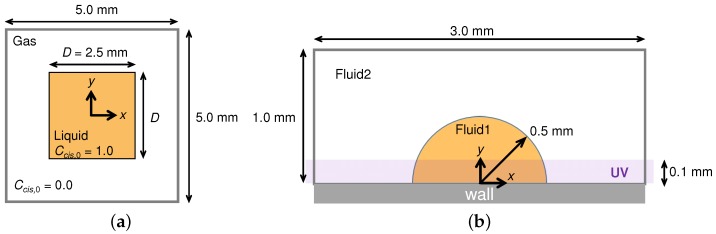
(**a**) Square liquid droplet surrounded by gas without gravity. Initially, the *cis* isomer ($C_{cis}=1$) dominates in the entire droplet. (**b**) Semisphere droplet on a flat wall to be partially irradiated with UV light that induces *cis-trans* photoisomerization. Initially, the *trans* isomer ($C_{cis}=0$) dominates in the entire domain. In this preliminary test, the *trans*-isomer liquid is assumed to have a contact angle of $\vartheta_{trans}=90^{\circ }$, and a UV-light irradiation area is limited only in the vicinity of the wall.

**Figure A2:**
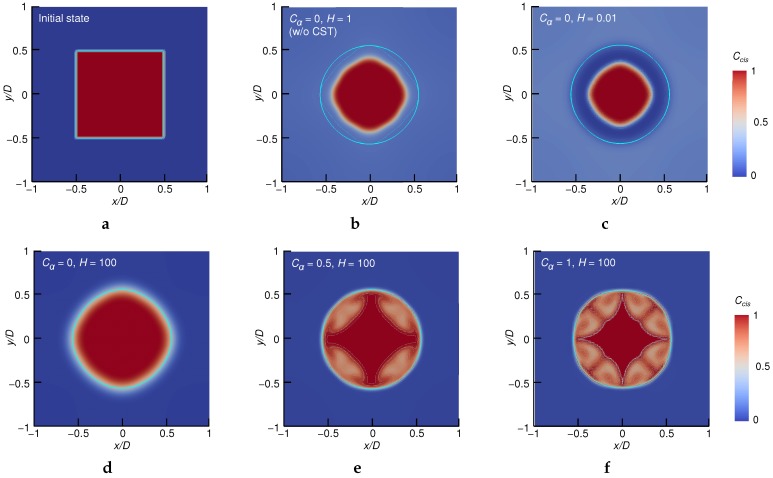
Contour of $C_{cis}$ distribution obtained at 0.2 s (except for **a**) after the release of a free square droplet, for different values of *H* and $C_{\alpha }$ and with/without the CST model. (**a**) Initial state of all cases at 0 s. (**b**) $C_{\alpha }=0$, H=1, without the CST method. (**c**) $C_{\alpha }=0$, H=0.01. (**d**) $C_{\alpha }=0$, H=100. (**e**) $C_{\alpha }=0.5$, H=100. (**f**) $C_{\alpha }=1$, H=100. Solid cyan line outlines the droplet shape.

**Figure A3:**
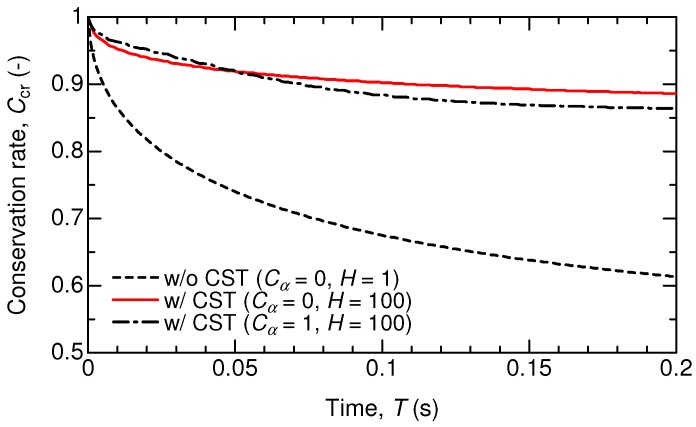
Comparison of the temporal variation of conservation of $C_{cis}$ in the liquid.

**Figure A4:**
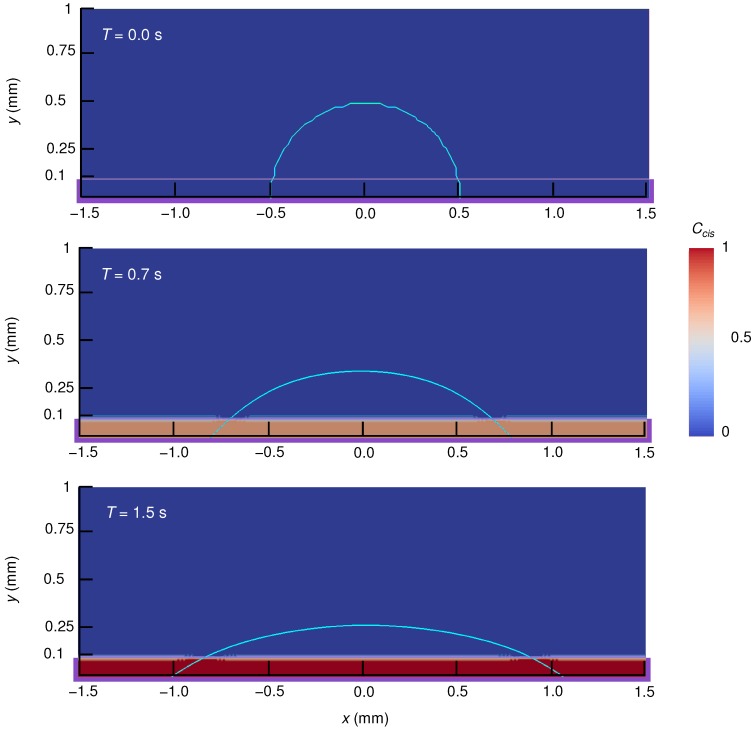
Temporal evolution of a droplet due to the photoisomerization-induced contact-angle change. Contour shows $C_{cis}$. The contact angles for $C_{cis}=0$ and 1 are $30^{\circ }$ and $90^{\circ }$, respectively. Solid cyan line outlines the droplet shape, and the purple-colored regions represent the UV-light irradiation area.

**Figure A5:**
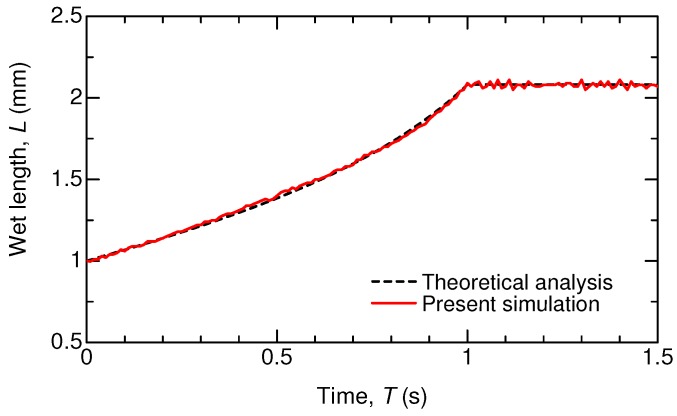
Temporal variation of the wet length of the droplet with the *cis-trans* photoisomerization by UV-light irradiation. The red line is a numerical result, while the black dashed line is a theoretical solution expressed by Equation (A2).

## Appendix A. Preliminary Tests

The present simulations were executed in a framework of a customized opensourse solver in OpenFOAM${}^{®}$ [29]. By tuning the solver code, we combined several schemes of the CST model and the CSF model to obtain sharp liquid–air interfaces and to suppress scalar mass flux across the interfaces. For those models, we should determine the optimal values of the relevant numerical parameters. In particular, the Henry coefficient *H* in Equation (17) and $C_{\alpha }$ in Equation (6) are crucial parameters. As a preliminary test to examine *H* and $C_{\alpha }$, which provide a reasonable result for the presently-given combination of fluids and scales, numerical simulations were carried out on a square droplet. In addition, the solver code was extended to simulate the photoisomerization in a part irradiated with UV light. To validate our code in terms of the modelling of the photoisomerization, we simulated a droplet on a flat plate, where the relationship between the wetting width and the contact angle is known theoretically. Figure A1 schematically illustrates the configurations of two preliminary simulations, the results of which will be reported in the following subsections.

### Appendix A.1. Scalar Transport Near the Interface

A square droplet with a length of *D* = 2.5 mm was given in the center of a computational domain: see also Figure A1a. The computational domain size and the number of grids were $L_{x}=L_{y}=5$ mm and $N_{x}=N_{y}=100$, respectively. The surface tension was fixed at $\sigma_{\mathrm{trans}}=40$ mN/m, since no UV-light irradiation was considered here. Other physical properties were the same as the liquid column driven by the photoisomerization, as given in Table 1. By systematically changing the Henry coefficient to H=0.01, 1, and 100, and the interface compression value to $C_{\alpha }=0$, 0.5, and 1, we investigated the degree of leakage of $C_{cis}$ through the liquid–air interface and the influence of spurious currents occurring near the interface.

Figure A2 shows the cross-sectional views of the droplet, where the interface has been deformed into the circle shape by the surface tension. In the simulation without the CST model, we detected a severe leakage of the *cis* isomer from the droplet, as shown in (b). Figure A2c also shows a significant decrease in $C_{cis}$ inside the droplet. By applying the CST model with a large Henry coefficient H=100, we successfully avoid an increase in $C_{cis}$ outside the droplet, as visualized in (d)–(f). Non-zero $C_{\alpha }$ values may provide more suppression of variations in $C_{cis}$ outside of the droplet, but the $C_{cis}$ conservation inside the droplet would obviously suffer from the spurious currents that exist near the liquid–air interface, as shown in (e) and (f).

In order to confirm the suppression of leakage from the droplet by employing the CST model with $C_{\alpha }=0$ and H=100, we define an index representing the conservation of $C_{cis}$ in the liquid. The following index $C_{\mathrm{cr}}$ is a ratio between an arbitrary time *T* and the initial t=0, regarding the volume-integrated *cis*-isomer concentration:

(A1) $$C_{\mathrm{cr}}=\frac{{\left[\int_{A}C_{cis}\cdot \alpha dA\right]}_{t=T}}{{\left[\int_{A}C_{cis}\cdot \alpha dA\right]}_{t=0}}.$$

Here, *A* represents the area of the computational domain, since the present simulation was performed in two-dimensional space. This ratio $C_{\mathrm{cr}}$ would be time-dependent in simulations, once a leakage of $C_{cis}$ from the liquid to air phase occurs. In the ideal situation, $C_{\mathrm{cr}}$ should be 1 at any time. As shown in Figure A3, the case visualized also in Figure A2d indeed exhibits less deviation from the unity value. According to this result, we have chosen the CST model with $C_{\alpha }=0$ and H=100 for the main liquid-column simulation.

### Appendix A.2. Changed Static Contact Angle by Photoisomerization

An initially-hemispherical droplet with a radius of $R_{0}=0.5$ mm was placed on the wall, as shown in Figure A1b. The computational domain size and the number of grids were $L_{x}\times L_{y}=3\times 1$ mm${}^{2}$ and $N_{x}\times N_{y}=150\times 50$, respectively. The surface tension was fixed at $\sigma_{\mathrm{trans}}=40$ mN/m without any change due to photoisomerization, but the static contract angle ranged from $\vartheta_{trans}=90^{\circ }$ to $\vartheta_{cis}=30^{\circ }$. The reaction rate constant in this droplet simulation was set at a rather large value of k=1 s${}^{-1}$. Other physical properties were the same as the main liquid-column simulation, as shown in Table 1.

Figure A4 shows the cross-sectional views of the droplet, where the contact angle between the liquid–air interface and the bottom solid wall can be observed to change gradually from the given $\vartheta_{trans}$ to $\vartheta_{cis}$. Such a decrease in the contact angle can be interpreted as an increase in the wettability of the liquid.

The droplet wet length *L* can be derived theoretically from the initial droplet size $R_{0}$ and the contact angle ϑ. By assuming that the inertia should be negligible compared to the surface tension and viscosity, the expression of

(A2) $$L=2R_{0}\sin\vartheta \sqrt{\frac{\pi }{2\left(\vartheta -\sin\vartheta \cos\vartheta \right)}}$$

would be valid. In the present (preliminary) system, ϑ should be changed monotonically from that for the *trans* isomer to that for the *cis* isomer as a function of $C_{cis}$, which is also time dependent in the UV-irradiated area and can be estimated mathematically on the basis of Equation (18). In Figure A5, the predicted evolution of the wet length is plotted with a dashed line. At T=1 s, $C_{cis}$ in the light-irradiated liquid has attained $C_{cis}=1$; that is, a state of all *cis* isomers in the vicinity of the wall. After that, no expansion of the wet length occurs and the droplet maintains the contact angle of $\vartheta =\vartheta_{cis}$. Also shown in Figure A5 is the result obtained by the present simulation. It can be confirmed that the present simulation reasonably demonstrates the temporal variation of the wet length, implying the validity of our simulation code with respect to the photoisomerization and its accompanying variation of the wettability.
